# Magneto-Immunoassay for the Detection and Quantification of Human Growth Hormone

**DOI:** 10.3390/bios12020065

**Published:** 2022-01-25

**Authors:** Almira Ramanaviciene, Anton Popov, Ema Baliunaite, Benediktas Brasiunas, Asta Kausaite-Minkstimiene, Ugur Tamer, Gailute Kirdaite, Eiva Bernotiene, Ali Mobasheri

**Affiliations:** 1 Department of Immunology, State Research Institute Centre for Innovative Medicine, LT-08406 Vilnius, Lithuania; anton.popov@imcentras.lt (A.P.); asta.minkstimiene@imcentras.lt (A.K.-M.); 2 Nanotechnas—Center of Nanotechnology and Materials Science, Faculty of Chemistry and Geosciences, Institute of Chemistry, Vilnius University, LT-03225 Vilnius, Lithuania; emabaliunaite@gmail.com (E.B.); benediktas.brasiunas@chgf.vu.lt (B.B.); 3 Department of Analytical Chemistry, Faculty of Pharmacy, Gazi University, Ankara TR-06330, Turkey; utamer@gazi.edu.tr; 4 Department of Experimental, Preventive and Clinical Medicine, State Research Institute Centre for Innovative Medicine, LT-08406 Vilnius, Lithuania; gailute.kirdaite@imcentras.lt; 5 Department of Regenerative Medicine, State Research Institute Centre for Innovative Medicine, LT-08406 Vilnius, Lithuania; eiva.bernotiene@imcentras.lt (E.B.); ali.mobasheri@oulu.fi (A.M.); 6 Department of Chemistry and Bioengineering, The Faculty of Fundamental Sciences, Vilnius Gediminas Technical University, Vilnius-Tech, LT-10223 Vilnius, Lithuania; 7 Research Unit of Medical Imaging, Physics and Technology, Faculty of Medicine, University of Oulu, FI-90014 Oulu, Finland; 8 Departments of Orthopedics, Rheumatology and Clinical Immunology, University Medical Center Utrecht, 508 GA Utrecht, The Netherlands; 9 Department of Joint Surgery, The First Affiliated Hospital, Sun Yat-sen University, Guangzhou 510080, China; 10 World Health Organization Collaborating Center for Public Health Aspects of Musculoskeletal Health and Aging, Université de Liège, 4000 Liège, Belgium

**Keywords:** human growth hormone, gold shell magnetic nanoparticles, sandwich-type colorimetric magneto-immunoassay

## Abstract

Physiological and endocrine maintenance of a normal human growth hormone (hGH) concentration is crucial for growth, development, and a number of essential biological processes. In this study, we describe the preparation and characterization of magnetic nanoparticles coated with a gold shell (MNPs-Au). The optimal surface concentration of monoclonal anti-hGH antibodies (m-anti-hGH) on magnetic nanoparticles, as well as conditions that decrease non-specific interactions during the magneto-immunoassay, were elaborated. After the selective recognition, separation, and pre-concentration of hGH by MNPs-Au/m-anti-hGH and the hGH interaction with specific polyclonal biotin-labeled antibodies (p-anti-hHG-B) and streptavidin modified horseradish peroxidase (S-HRP), the MNPs-Au/m-anti-hGH/hGH/p-anti-hGH-B/S-HRP immunoconjugate was formed. The concentration of hGH was determined after the addition of 3,3′,5,5′-tetramethylbenzidine and hydrogen peroxide substrate solution for HRP; the absorbance at 450 nm was registered after the addition of STOP solution. The developed sandwich-type colorimetric magneto-immunoassay is characterized by a clinically relevant linear range (from 0.1 to 5.0 nmol L^−1^, *R*^2^ 0.9831), low limit of detection (0.082 nmol L^−1^), and negligible non-specific binding of other antibodies or S-HRP. The obtained results demonstrate the applicability of the developed magneto-immunoassay for the concentration and determination of hGH in the serum. Additionally, important technical solutions for the development of the sandwich-type colorimetric magneto-immunoassay are discussed.

## 1. Introduction

Nanotechnology had a profound impact on different fields of research, including analytical systems for sensitive, selective, quick, and user-friendly detection of medically important biomolecules in the blood. Scientists face a great challenge when it comes to the detection of an ultralow concentration of the biomarkers in a relatively large sample volume. An effective strategy to solve this problem is the application of functionalized magnetic nanoparticles (MNPs) or magnetic beads (MBs) for the selective recognition, separation, and pre-concentration of the desired analyte from the sample. Metals (iron, cobalt, nickel), metal oxides (iron oxides and ferrites), or ferromagnetic alloy MNPs and MBs are characterized by a large active surface area for the immobilization of specific antibodies or receptors, the ability to maintain their magnetic properties after modification by biomolecules, and by the rapid separation of analyte from the mixture using an external magnetic field [1,2,3]. The increased sensitivity of analytical systems using different immunoassay formats and various techniques, the reduction in non-specific binding effect, and a shorter response time are major advantages rendered by the use of MNPs [4]. Improvement in the detection of circulating tumor cell phenotypes [5], microorganisms [6], nucleic acids [7], proteins [8], and small molecules [9] using MNPs was experimentally confirmed. Furthermore, MNPs serve for the separation and pre-concentration of different biomarkers or toxins present in the same sample, followed by the multiplex analysis using specific luminescent tags. Inorganic, semiconductor nanocrystals, quantum dots (QDs), immobilized on the detection antibody, are an ideal tag for the multiplex binding event evaluation and multiple biomarkers detections [10,11]. The excited QDs exhibit size and composition-dependent fluorescence emission spectra, allowing simultaneous detection of different biomarkers [12].

The concentration of hormones that are important for normal physiological functions is very low in blood and serum samples. The normal range for human growth hormone (hGH) (also known as somatotropin), one of several hormones secreted by the anterior pituitary gland in the brain, depends on gender and age and typically ranges from 0.4 ng mL^−1^ (0.018 nmol L^−1^) to 50 ng mL^−1^ (2.273 nmol L^−1^) [13]. hGH is a polypeptide hormone consisting of various circulating isoforms, between which the largely prevalent (~50%) is a 22 kDa hGH isoform consisting of 191 amino acids. The second most abundant form is 20 kDa hGH, only making up 5–9%. The rest of hGH is found in dimer and oligomer forms or as hGH fragments [14,15,16]. The normal concentration of hGH is essential for regular body growth and a number of biological processes, such as the metabolism of lipids, carbohydrates, and proteins [17]. An excess of this hormone was determined to cause gigantism [18], insulin resistance [19], and finally, diabetes [20], while deficiency results in growth retardation in children and deficiency syndrome in adults [21]. Thus, the determination of both the deficiency or excess of hGH in serum is very important for the evaluation of disorders in this hormone secretion and for the identification of various disorders that occur in people of different ages.

Different format immunoassays and various immunosensors based on different signal transducers were developed for the determination of hGH distinguished by the low limit of detection (LOD) and optimal linear range in a specific case, namely, standard double-antibody radioimmunoassay and immunoradiometric assay [22], immunofluorometric assay [23], immunochemiluminescent assay [24], sandwich enzyme-linked immunoassay (quantitative) (working range 2.5–600 pg mL^−1^, sensitivity 4 pg mL^−1^) [13], direct detection in serum using surface plasmon resonance (SPR) immunosensors based on the surface modified by site-directed oriented reduced half antibody fragments (LOD 0.0034 μmol L^−1^, linear range 0.01–0.72 μmol L^−1^) and whole antibody via protein G (LOD 0.99 nmol L^−1^, linear range 3–9 nmol L^−1^) [25,26], electrochemical impedance spectroscopy immunosensors (LOD 0.64 pg mL^−1^, linear range 3–100 pg mL^−1^) [27], and disposable electrochemical magneto-immunosensor (LOD 0.005 ng mL^−1^, linear range 0.01–100 ng mL^−1^) [28].

In this study, we describe the preparation of MNPs coated with gold shell (MNPs-Au) and modified with monoclonal anti-hGH antibodies (m-anti-hGH) for the application in a magneto-immunoassay for the detection of hGH. These MNP-Au nanoparticles were used for the selective recognition, separation, and pre-concentration of hGH from a sample and for further determination in a small volume by sandwich-type magneto-immunoassay using specific polyclonal biotin-labeled antibodies (p-anti-hHG-B), horseradish peroxidase modified with streptavidin (S-HRP), 3,3′,5,5′-tetramethylbenzidine (TMB), and hydrogen peroxide substrate solution for HRP. Technical solutions for the development of magneto-immunoassay were discussed. The performance of the developed colorimetric magneto-immunoassay for the detection and quantification of low concentrations of hGH in relatively large volume samples was evaluated. The further applications of the developed magneto-immunoassay were discussed.

## 2. Materials and Methods

### 2.1. Materials and Reagents

Recombinant human growth hormone from E. coli (hGH), mouse monoclonal anti-human growth hormone IgG2B antibodies (m-anti-hGH), and goat polyclonal anti-human growth hormone biotinylated IgG antibody (p-anti-hGH-B) were obtained from R&D Systems (Minneapolis, MN, USA). Steptavidin-HRP conjugate (S-HRP) and 3,3′,5,5′-tetramethylbenzidine (TMB) and hydrogen peroxide substrate for HRP, and 0.5 mol L^−1^ H_2_SO_4_ (STOP solution) were acquired from ELISA kit produced by BioVendor (Brno, Czech Republic). N-(3-dimethylaminopropyl)-N-ethylcarbodiimide hydrochloride (EDC), N-hydroxysuccinimide (NHS), methanol, hexadecyltrimethylammonium bromide (CTAB), and PBS tablets (0.01 M phosphate-buffered saline, pH 7.4) were purchased from Carl Roth (Karlsruhe, Germany). Hydrogen tetrachloroaurate trihydrate (HAuCl_4_·3H_2_O) was received from Alfa Aesar (Karlsruhe, Germany). The 11-mercaptoundecanoic acid (11-MUA), perchloric acid, iron (II) sulfate heptahydrate (FeSO_4_·7H_2_O), and bovine serum albumin (BSA) were received from Sigma-Aldrich (Steinheim, Germany). Iron (III) chloride hexahydrate (FeCl_3_·6H_2_O) and ethylenediaminetetraacetic acid (EDTA) were obtained from AppliChem (Karlsruhe, Germany). Ethanol was acquired from Honeywell (North Carolina, USA), sodium borohydride (NaBH_4_) was from Merck (Darmstadt, Germany), and hydroxylamine hydrochloride was from Lach-Ner (Neratovice, Czech Republic). All aqueous solutions were prepared in ultrapure deionized water.

### 2.2. Synthesis of Magnetic Nanoparticles

The synthesis of magnetic gold-coated nanoparticles consisted of two parts—iron oxide (Fe_3_O_4_) MNPs synthesis and gold shell coating procedure. MNPs were synthesized using the co-precipitation method according to the already published protocol [29,30]. Briefly, 125 mL of 1 mol L^−1^ NaOH solution was added dropwise under vigorous stirring into 10 mL of the solution consisting of 1.28 M FeCl_3_ and 0.64 mol L^−1^ FeSO_4_. The formed precipitate was collected with the help of a magnet, washed three times with deionized water, and then kept in 2 mol L^−1^ HClO_4_ solution overnight under argon (Elme Messer Gaas, Lithuania) atmosphere to obtain Fe_3_O_4_ nanoparticles. The color of MNPs becomes brown. Subsequently, the nanoparticles were collected by centrifugation at 12,000× *g* for 20 min and washed three times with deionized water and once with ethanol, and finally, were left to dry in air.

### 2.3. Coating Magnetic Nanoparticles with a Gold Shell

Firstly, 5 mg of dried magnetic nanoparticles were sonicated in 5 mL of water until fully dispersed. Then, 10 mL of 0.27 mol L^−1^ EDTA solution prepared in 1 mol L^−1^ NaOH was added, and MNPs were re-suspended by using an ultrasonic bath. Particles were collected with a magnet and dispersed in a 10 mL mixture of 0.1 mol L^−1^ CTAB and 0.01 mol L^−1^ HAuCl_4_ solution. Subsequently, the 150 mg hydroxylamine hydrochloride was added to the vigorously stirred solution in order to reduce AuCl_4_^−^ ions to Au(0) on the surface of MNPs. The color of the solution changed from brown to dark red, indicating the formation of nanoparticles with gold shells (MNPs-Au). These particles were characterized by UV-vis spectrophotometer Lambda 25 (Perkin Elmer, Shelton, WA, USA) and transmission electron microscope (TEM) Tecnai F20 X-TWIN (Eindhoven, The Nederland). X-ray diffraction (XRD) measurements were performed using a MiniFlex II diffractometer (Rigaku, Japan). The diffractograms were recorded in the 2θ range from 25° to 80° using CuK_α_ λ = 1.5406 Å radiation.

### 2.4. Modification of MNPs-Au by m-Anti-hGH Antibodies

**Figure 1:**
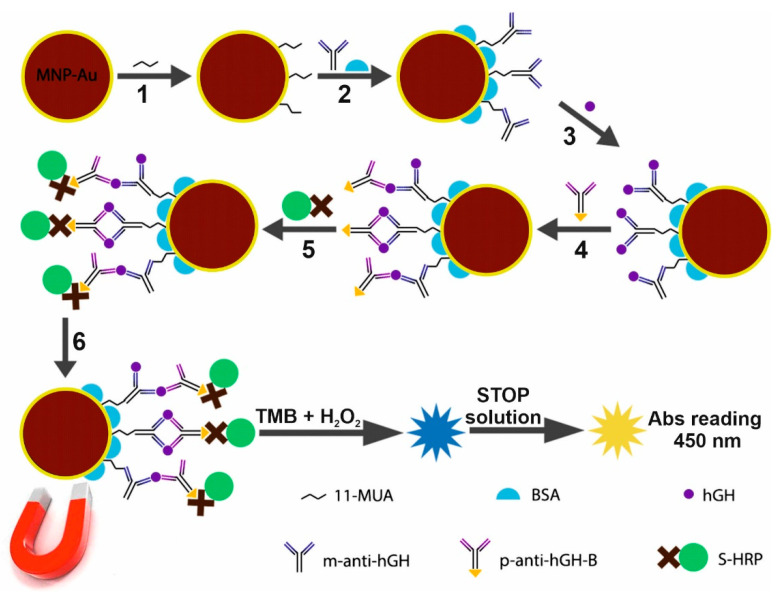
Schematic illustrating the design of the sandwich-type magneto-immunoassay for the detection of human growth hormone (hGH). The magnet was used in all steps for the collection of modified MNPs-Au.

In order to be able to immobilize m-anti-hGH antibodies successfully, the CTAB used in the synthesis procedure of the particles first had to be removed from the MNPs-Au surface [31]. Briefly, 800 µL of 30 mmol L^−1^ NaBH_4_ was added to 1.6 mL 0.2 mg mL^−1^ MNPs-Au solution, and the mixture was stirred for 1 h. CTAB-free nanoparticles were washed with deionized water and further used for covalent immobilization of antibodies. Firstly, a self-assembled monolayer (SAM) was formed by keeping MNPs-Au in a 1 M 11-mercaptoundecanoic acid (11-MUA) solution for 2 h (Figure 1, step 1). After washing with H_2_O, the carboxyl groups of 11-MUA were activated with a mixture consisting of 200 mmol L^−1^ EDC and 50 mmol L^−1^ NHS for 15 min. Magnetically collected MNPs-Au were added to the solutions consisting of different concentrations of m-anti-hGH antibodies (200, 330, 660, and 984 nmol L^−1^). After 2 h MNPs-Au/m-anti-hGH particles were washed three times with 10 mM PBS solutions (pH 7.4) and kept in a solution of 1 % BSA made in 10 mmol L^−1^ PBS (pH 7.4) for 1 h at room temperature and overnight at +4 °C to block the unreacted activated esters and the free surface (Figure 1, step 2).

Optimal m-anti-hGH concentration was determined by keeping MNPs-Au, which were modified with different m-anti-hGH concentrations, in solutions of 400 nmol L^−1^ hGH and 990 nmol L^−1^ p-anti-hGH-B, respectively. After the interaction with hGH and p-anti-hGH-B, immunoconjugates were washed three times using a 0.1% BSA solution in 10 mmol L^−1^ PBS (pH 7.4) and then left in 100 μL solution of S-HRP for 30 min. Another washing step was performed using 10 mmol L^−1^ PBS solution, and then a 100 μL of TMB substrate was added. An enzymatic reaction lasted for 10 min in the dark and was stopped by adding 100 μL of STOP solution. The absorbance of the formed yellow product was registered at 450 nm. After determining the optimal m-anti-hGH concentration for MNPs-Au modification, the analytical system based on the application of MNPs-Au for the collection and pre-concentration of hGH was designed.

### 2.5. Development of Sandwich Type Magneto-Immunoassay for hGH Detection Using MNPs-Au

An amount of 0.2 mg mL^−1^ of MNPs-Au was first modified using NaBH_4_, then a SAM of 11-MUA was formed, and m-anti-hGHs at optimal concentration were covalently immobilized onto the MNPs-Au surface as described previously (Figure 1, steps 1–2). Afterward, MNPs-Au/m-anti-hGH interacted with different concentrations of hGH (Figure 1, step 3), followed by interaction with 100 nM p-anti-hGH-B (Figure 1, step 4) and S-HRP (Figure 1, step 5). The solution of 0.1% BSA in 10 mmol L^−1^ PBS (pH 7.4) was used for washing three times after each step of the interaction. After carrying out the enzymatic HRP reaction and stopping this reaction with STOP solution, the absorbance of the formed yellow product was registered at 450 nm after the removal of immunoconjugates from the solution. The scheme of magneto-immunoassay for the detection of hGH is provided in Figure 1. It should be mentioned that in order to reduce the non-specific adsorption of proteins and MNPs-Au surface, test tubes were coated with BSA using 1% solution for 1 h at room temperature and overnight at +4 °C.

## 3. Results and Discussion

### 3.1. Characterization of Magnetic MNPs-Au

**Figure 2:**
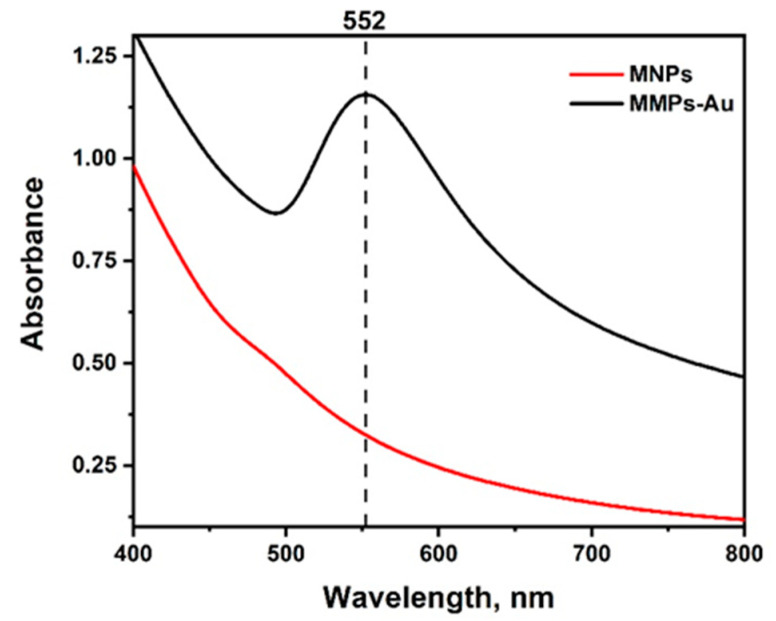
Absorbance spectra of magnetic nanoparticles (MNPs) before and after coating with the gold layer (MNPs-Au).

A two-stage synthesis procedure was used for the preparation of MNPs-Au. The synthesized iron oxide nanoparticles were coated by a gold-layer shell. In order to demonstrate the formation of the gold layer around MNPs, UV-vis spectra were registered (Figure 2).

Absorption spectra for Fe_3_O_4_ nanoparticles [32], as expected, did not produce an absorption peak in the visible spectrum range. However, after the synthesis of a gold shell, a characteristic surface plasmon band with a maximum of 552 nm can be observed corresponding to the local surface plasmon resonance (LSPR) of spherical gold nanoparticles, confirming successful coating of MNPs by a gold layer [33]. Quite a high intensity of LSPR band can be explained by high extinction coefficient, which is characteristic for bigger gold nanoparticles, wherein broad band indicates polydispersity of the gold coatings [34].

**Figure 3:**
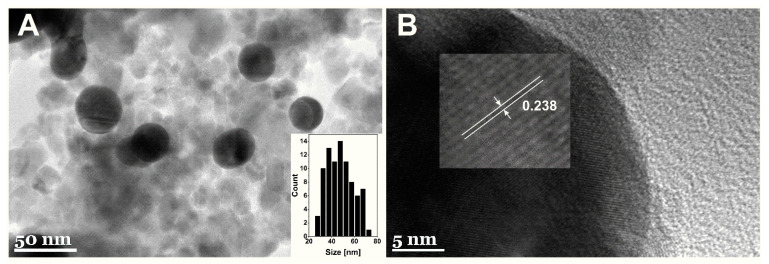
TEM images of synthetized MNPs-Au under different magnification. Insets: (**A**) the particle size distribution histogram and (**B**) HRTEM image demonstrating the Au lattice spacing.

The TEM micrographs of MNPs-Au are given in Figure 3. Spherical gold-coated nanoparticles surrounded by non-coated magnetic particles were observed. MNPs-Au appears darker in comparison with non-coated particles. This difference can be associated with higher electron density for gold versus iron oxide [29]. These results coincide with previously published work by Tamer et al. [35]. The average diameter of synthesized MNPs-Au was equal to 47.6 ± 11.3 nm (Figure 3A). Additionally, it was found that the lattice fringes taken from the surface of MNPs-Au showed 2.38 A interplanar spacing (Figure 3B) attributed to the fcc-structured gold (111) plane, further confirming the formation of a gold shell on the surface of magnetic nanoparticles.

**Figure 4:**
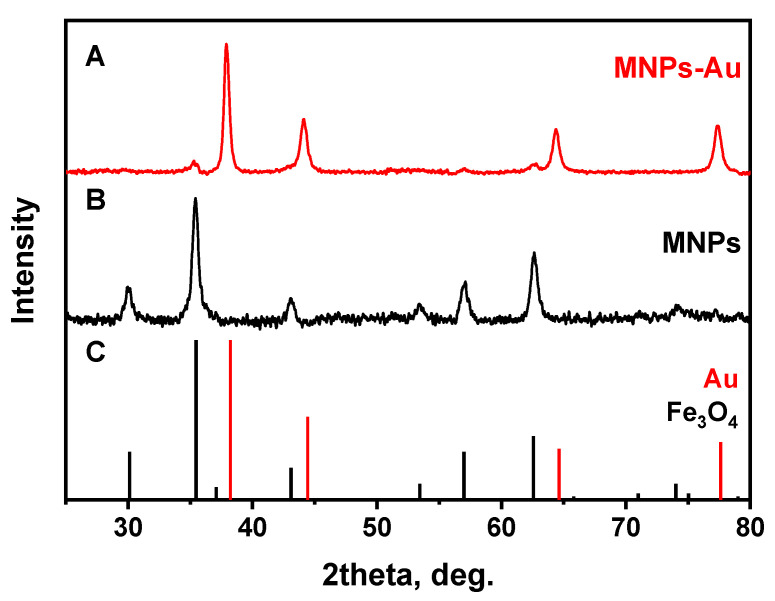
XRD patterns of (**A**) MNPs-Au, (**B**) MNPs, and (**C**) standards of Au (ICDD 00-004-0784) and Fe_3_O_4_ (ICDD 00-019-0629).

The crystalline structure of synthesized nanoparticles before and after the coating was examined by XRD analysis. The XRD patterns for MNPs and MNPs-Au are presented in Figure 4. It was shown that in the case of MNPs, magnetite (Fe_3_O_4_) nanoparticles were synthesized. All diffraction peaks match very well with the standard XRD data (ICDD 00-019-0629). As a result of the gold layer coating, the peaks at 38.12°, 44.14°, 64.4°, and 77.38° were observed, which are assigned, respectively, to (111), (200), (220), and (311) reflections of the face-centered cubic structure of metallic gold. It can be observed that (111) plane is the predominant orientation. Some Fe_3_O_4_ peaks are also seen in the XRD pattern of MNPs-Au. The low intensity of these peaks and the absence of other magnetite peaks can be explained by a heavy atom effect of Au [32].

### 3.2. Magneto-Immunoassay Performance Optimization

The whole procedure for the hGH detection, involving the m-anti-hGH immobilization onto MNPs-Au surface; the interaction with hGH, p-anti-hGH-B, and S-HRP; and followed by the enzymatic reaction and the evaluation using microplate spectrophotometer SpectraMax i3 (Molecular Devices, San Jose, CA, USA), is schematically presented in Figure 1. The first step of this immunoassay optimization involves the selection of the optimal MNPs-Au and m-anti-hGH concentrations ratio.

**Figure 5:**
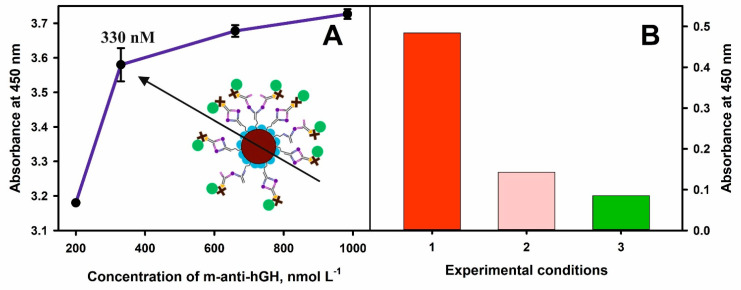
(**A**) Dependence of the optical response at 450 nm on the concentration of m-anti-hGH used for covalent immobilization onto MNPs-Au. (**B**) The reduction in the non-specific interactions during the magneto-immunoassay. The absorbance of the blank samples: 1—using only 10 mmol L^−^^1^ PBS; 2—after blocking test tubes walls with 1% BSA in 10 mmol L^−^^1^ PBS; 3—after blocking of test tubes walls with 1% BSA in 10 mmol L^−^^1^ PBS and using dilution and washing solutions consisting of 0.1% BSA in 10 mmol L^−^^1^ PBS.

Thus, in all experiments, 0.2 mg mL^−1^ concentration of MNPs-Au and 200, 330, 660, and 980 nmol L^−^^1^ concentrations of m-anti-hGH were used for the covalent immobilization onto nanoparticles modified with 11-MUA. Additionally, the 400 nmol L^−^^1^ of hGH and 990 nmol L^−^^1^ of p-anti-hGH-B were used in this experiment (Figure 5A). The affinity interactions and immunoconjugate composed of MNPs-Au/m-anti-hGH/hGH/p-anti-hGH-B/S-HRP formation were monitored by the addition of TMB substrate followed by the enzymatic reaction product formation and absorbance registration after the addition of the STOP solution. The absorbance at 450 nm increased by 0.4 when the concentration of m-anti-hGH was increased from 200 to 330 nmol L^−^^1^. By further increasing the concentration of m-anti-hGH to 660 and 990 nmol L^−^^1^, the absorbance increased by 0.1 and 0.5, respectively. Based on the obtained experimental results and taking into account observations by other authors, better results of analytical systems are achieved using a smaller surface concentration of antibodies [36]; for further experiments, 330 nmol L^−^^1^ concentration of m-anti-hGH was selected. Additionally, the concentration of p-anti-hGH-B was reduced to 100 nmol L^−^^1^ in order t to detect lower hGH levels and reduce the non-specific binding of these antibodies.

The dilution and washing solution consisting of 0.1% BSA in 10 mmol L^−^^1^ PBS (pH 7.4) was used to reduce non-specific binding during the indirect detection of hGH. Furthermore, the test tubes were modified with 1% BSA in 10 mmol L^−^^1^ PBS (pH 7.4) for 1 h. This blocking procedure helped to reduce the absorbance of the blank sample by 5.7 times (Figure 5B). This result is explained by the decrease in the level of non-specific binding of p-anti-hGH and S-HRP. Therefore, the background signal was lowered and subtracted from the experimental results with hGH, and the selected experimental conditions allowed to determine hGH at low concentrations accurately.

### 3.3. Analytical Characteristics of Sandwich Type Magneto-Immunoassay for hGH Detection Using MNPs-Au

**Figure 6:**
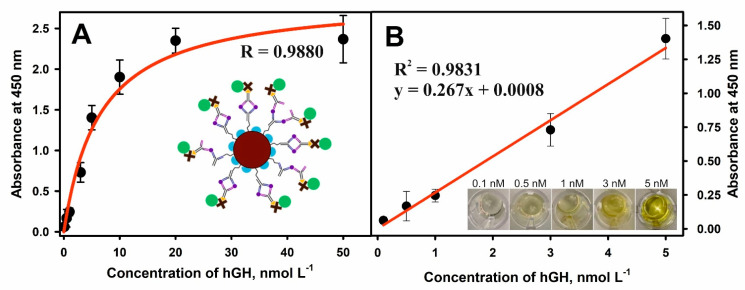
(**A**) Dependence of absorbance at 450 nm on the concentration of hGH. (**B**) The linear range of the developed sandwich-type magneto-immunoassay for hGH detection using MNPs-Au. Conditions: 0.2 mg mL^−1^ of MNPs-Au modified with 330 nmol L^−1^ concentration of m-anti-hGH; 100 nmol L^−1^ concentration of p-anti-hGH-B; 100 μL solution of S-HRP; 100 μL of TMB substrate; 0.1–5.0 nmol L^−1^ concentrations of hGH.

An optical sandwich-type magneto-immunoassay based on the antibody-modified MNPs-Au application for the pre-concentration of hGH present in the solution was developed and optimized. The principal scheme and each step of sandwich magneto-immunoassay followed by the formation of the color enzymatic reaction product is explained in Figure 1. The MNPs-Au/m-anti-hGH were exposed to hGH concentrations of 0.01 to 50 nmol L^−^^1^, keeping constant concentrations of other reagents and the duration of the enzymatic reaction. The dependence of absorbance at 450 nm on different concentrations of hGH under optimized conditions is depicted in Figure 6A. The hyperbolic relationship between absorbance and hGH concentrations was obtained (*R* = 0.9880). The linear dependence (Figure 6B) was registered from 0.1 to 5.0 nmol L^−^^1^ concentrations of hGH (*R*^2^ = 0.9831). The LOD defined as the lowest concentration of hGH, which gives an analytical signal greater than the background plus 3 *σ*, was calculated to be 0.082 nmol L^−^^1^. The developed magneto-immunoassay is suitable for the determination of hGH in the normal hGH range and at higher concentrations than using standard sandwich-format ELISA. Additionally, LOD was improved from 2.2 times if compared with inhibition SPR-based immunosensor format [37] to 41.6 times [25] if compared with results obtained using direct SPR-based immunosensor format (Table 1).

The described system was also used with other non-hGH specific biotinylated antibodies to test the influence of non-specific binding by the affinity interaction with S-HRP. An amount of 5 nmol L^−1^ of hGH and 100 nmol L^−1^ of biotinylated monoclonal mouse antibodies against human cartilage oligomeric matrix protein (clone: 16F12) were used. In this case, the absorbance increased only by 1.7% compared to the control without any hGH, but in the presence of 100 nmol L^−1^ p-anti-hGH-B in the tested analytical system indicating low non-specific interaction for other biotinylated antibodies.

### 3.4. Determination of hGH in Spiked Serum Samples

In order to demonstrate the utility of the developed sandwich-type magneto-immunoassay using modified MNPs-Au for the detection of hGH in real samples, a human serum sample spiked with 3.5 nmol L^−1^ of hGH was tested. In order to evaluate the impact of the matrix and the possible presence of hGH on the analytical signal, a calibration curve with a known hGH concentration in the serum was constructed. We found the recovery of 3.5 mol L^−1^ of hGH to be 94.9%. Despite a slightly higher concentration of the determined hGH, these results demonstrate the applicability of the developed magneto-immunoassay for the analysis of hGH in real samples of small volume.

## 4. Conclusions

The colorimetric magneto-immunoassay for the determination and quantification of low concentrations of hGH was developed and evaluated. MNPs-Au modified with monoclonal anti-hGH antibodies were successfully applied for the separation of hGH from the relatively high volume sample and concentration in a low volume for further sensitive determination. The coating of MNPs with a gold shell simplifies the antibody immobilization step and allows different antibody immobilization methods to be used. Detection antibody–HRP conjugates were essential for the hGH detection, followed by the analytical signal amplification and registration. Many efforts are now being made to develop methods for the simultaneous detection of multiple biomarkers in a single sample. The proposed magneto-immunoassay methodology can be adapted for this purpose. We can achieve this goal by modifying MNP-Au with antibodies specific for various biomarkers; however, distinct signal amplification tags have to be applied, such as different enzymes or other materials. An ideal tag for the multiplex binding events evaluation and multiple biomarkers detections is QDs. The excited QDs exhibit size and composition-dependent fluorescence emission spectra, allowing simultaneous detection of different biomarkers. In summary, the developed and characterized magneto-immunoassay could be adapted to multiplex biomarker detection using different QDs characterized by a good quantum yield and high photochemical stability.

## Figures and Tables

**Table 1:** Comparison of analytical parameters for various formats of optical immunoassays and immunosensors used for hGH detection.

Analytical System for hGH Detection	Linear or Dynamic Range	LOD	Sensitivity	Ref.
SPR-based immunosensors for the direct detection using reduced half antibody fragments	10–720 nmol L^−1^	3.4 nmol L^−1^		[25]
SPR-based immunosensors for the direct detection using antibody immobilization via protein G	3–9 nmol L^−1^	0.99 nmol L^−1^		[26]
SPR-based inhibition immunosensor format using surface modified with hGH	18–542 ng mL^−1^ (0.82–24.6 nmol L^−1^)	4 ng mL^−1^ (0.18 nmol L^−1^)		[37]
Sandwich ELISA, p-anti-hGH/hGH/anti-hGH-B/S-HRP, absorbance at 450 nm.	1–25 ng mL^−1^ (0.046–1.14 nmol L^−1^)		0.25 ng mL^−1^ (0.0114 nmol L^−1^)	[38]
Sandwich ELISA, anti-hGH/hGH/anti-hGH-B/S-HRP, absorbance at 450 nm.	2.5–600 pg mL^−1^ 1.1·10^−4^–0.027 nmol L^−1^		4 pg mL^−1^ (1.8·10^−4^ nmol L^−1^)	[13]
Sandwich ELISA, m-anti-hGH/hGH/m-anti-hGH-HRP, absorbance at 450 nm.	0.5–50 ng mL^−1^ (0.023–2.27 nmol L^−1^)		0.5 ng mL^−1^ (0.023 nmol L^−1^)	[39]
Indirect detection, MNPs-Au/m-anti-hGH/hGH/p-anti-hGH-B/S-HRP immunoassay, absorbance at 450 nm.	0.1–5.0 nmol L^−1^	0.082 nmol L^−1^		Current work

## Data Availability

The data presented in this study are available on request from the corresponding author.
